# Is Blood Alcohol Level a Good Predictor for Injury Severity Outcomes in Motor Vehicle Crash Victims?

**DOI:** 10.1155/2011/616323

**Published:** 2011-09-14

**Authors:** Bikaramjit Mann, Ediriweera Desapriya, Takeo Fujiwara, Ian Pike

**Affiliations:** ^1^Department of Medicine, Faculty of Medicine, University of Calgary, 1403-29 Street NW, Calgary, AB, T2N 2T9, Canada; ^2^BC Injury Research and Prevention Unit, L408-4480 Oak Street, Vancouver, BC, V6H 3V4, Canada; ^3^Developemental Neurosciences and Child Health, Child and Family Research Institute, L408-4480 Oak Street, Vancouver, BC, Canada V6H 3V4; ^4^Department of Social Medicine, National Research Institute for Child Health and Development, Tokyo 1578535, Japan

## Abstract

Experimental studies in animals suggest that alcohol may influence pathophysiologic response to injury mechanisms. However, biological evidence for the alcohol-injury severity relationship provides conflicting results. The purpose of our retrospective cross-sectional study in 2,323 people was to assess whether blood alcohol level (BAC) adversely influences injury severity in victims of motor vehicle collisions (MVCs). We found no difference in mortality OR 1.09 (0.73–1.62), or length of hospital stay, and a trend for lower ISS score was found in the high-alcohol group (*P* = 0.052). Furthermore, the high-alcohol group demonstrated a lower adjusted rate of severe head injury OR 0.65 (0.48–0.87), chest injury OR 0.58 (0.42–0.80), and serious extremity injury OR 0.10 (0.01–0.76). The findings of our study do not demonstrate a dose-response relationship between alcohol consumption and injury severity in MVCs. This study implies that higher BAC may lead to less severe injuries, without impacting mortality or length of hospital stay, however, further research is required to elucidate the nature of this relationship.

## 1. Introduction

Acute and chronic alcohol consumption is a well-documented cause of substantial social burden to society as well as a strain on health care systems around the world [1]. In Canada, 88.6% of Canadians report drinking alcohol at least once during their lifetime, while 76.5% of Canadians report having used alcohol over the past 12 months, and the average age for beginning alcohol consumption is roughly 15.9 years [2]. Approximately, 5% of Canadians report heavy frequent drinking, which is defined as a person who drinks one or more times per week and consumes at least 5 or more drinks on each occasion [2]. While 36% of Canadians report themselves as light infrequent drinkers, defined as consuming less than 5 drinks on each drinking occasion and drinking less than once per week on average [2]. Policy makers have struggled with the acceptable limit of alcohol consumption and driving, particularly with zero-limit alcohol policies versus increased public education campaigns [3]. Adding to the complexity of this controversial public health issue is the uncertainty with respect to the strength of association between alcohol consumption and injury severity in the scientific literature.

Drivers who are intoxicated are more likely to be involved in motor vehicle collisions (MVCs) and those with a positive blood-alcohol-concentration (BAC) are more likely to be at fault in a collision [4, 5]. It has been suggested that impaired driving secondary to alcohol intoxication, and those with a positive BAC are also more likely to be fatally injured than drivers who are not under the influence of alcohol [6–10]. Some studies suggest that alcohol is associated with significantly increased injury severity and fatality, while other studies suggest that alcohol does not adversely affect the degree and clinical outcome of injury [11–25]. Although the association of alcohol consumption with increased risk of injury is well-documented in the literature based on controlled experimental and epidemiologic studies, it is controversial as to whether alcohol is associated with the severity and outcome of injury given that an injury occurs [26]. At least one explanation for the discrepancy in the literature has been attributed to selection bias resulting from triage and referral [26]. Other explanations that have been put forth include variability residing from potential confounders such as usual drinking patterns, risk-taking behaviour and substance use [27]. For instance, the combination of alcohol and benzodiazepines can have detrimental effects on driving beyond those of alcohol alone [28]. The objective of this study was to examine if alcohol consumption adversely affects the severity of injury in individuals involved in MVCs, which included drivers, passengers, and pedestrians.

## 2. Materials and Methods

In this retrospective cross-sectional observational study we collected data from the British Columbia Trauma Registry (BCTR) in the province of British Columbia (BC), Canada. We have used the *Strengthening the Reporting of Observational Studies in Epidemiology* (STROBE) statement as a guide for reporting this paper [29]. The BCTR collects and maintains data on all trauma patients admitted to any of the nine trauma receiving facilities in BC. The health records of trauma patients are flagged on admission or later by the staff. Trauma registry staff analyze the health records and enter data into the system using collector software. The BCTR contains information on the injury mechanism and is the most detailed source of information on severe injuries throughout BC [30]. We included patients who were older than 10 years of age, for the period January 1, 2003 to December 31, 2005, inclusive. Patients were included if they were admitted to the trauma facility within 7 days of sustaining injury, and admission to hospital, at least initially, was due to traumatic injury not underlying medical or psychiatric illness (except for patients suffering a spinal cord injury), requiring 2 or more days of hospitalization with an Injury Severity Score >12. All trauma-related deaths regardless of length of stay in hospital were also included in our analysis. Patients were excluded if they had been admitted for psychiatric purposes for self-inflicted injuries; drowning with no associated anatomical injuries; admitted for falls related to seizures, syncope, general debility, or weakness; chronic subdural or epidurals; pathologic fractures; cellulitis or infections related to complications of lacerations or animal bites; poisonings or overdoses; decompression sickness; fractures related to old or indeterminate causes related to a fall and foreign body in a hollow viscus. 

We separated our cohort into three categories: (1) no alcohol consumption, (2) low-alcohol consumption (1–17.3 mmol/L or <0.08 g/dL), and (3) high-alcohol consumption (>17.3 mmol/L or ≥0.08 g/dL), and we used the Injury Severity Score (ISS) as one of the outcome measures for our analysis, which is a widely accepted injury severity scale [26, 31]. To avoid misclassification of nonsevere, cases we used a ISS score >15 [32]. Our primary outcomes were mortality, length of hospital stay, ISS, head, chest, and extremity injuries. Secondary outcomes included situational factors such as collision time, time of day, season, and location of collision. Ethics approval for this study was granted from the Office of Research Services' Behavioral Research Ethics Board (BERB) at the University of British Columbia, Vancouver, BC, Canada. 

Characteristics of individuals and MVC are included in Table 1. We compared injury severity and mortality according to MVC locations, as well as examining different age groups. Logistic regression models were used to estimate age-associated risks while controlling for confounders such as gender and crash types. Characteristics of collision severity, situation, and injury information were compared between age groups using bivariate analyses. Logistic regression was used to analyze categorical variables, while *t*-tests were employed to analyze continuous variables. Significant factors in bivariate analyses were entered into multiple regression models to identify independent risk factors. Stata version 9.0 was used for all analyses, with statistical significance considered when *P* value <0.05.

## 3. Results and Discussion

Table 1 describes characteristics of the 2,323 patients included in our study. The no alcohol group comprised 62% (*N* = 1,445) of patients, while the low-alcohol and high-alcohol groups consisted of 12% (*N* = 273) and 26% (*N* = 605), respectively. Those <18 years of age were less likely to be in the high-alcohol group (*P* value <0.001). There was a greater proportion of men (*P* < 0.001), and more MVCs occurred at night (*P* < 0.001) on weekends, and the location of MVCs were more likely to occur on the street or highway (*P* < 0.001). Drivers were more likely to be injured than passengers or pedestrians (*P* = 0.002), and a greater proportion of MVCS were multiple vehicle crashes (*P* < 0.001). Although information with respect to seatbelt usage was missing in 16% of cases, those in the high-alcohol group were less likely to be wearing seatbelts (*P* < 0.001). 

For both the primary outcomes of mortality (*P* = 0.377) and length of hospital stay (*P* = 0.775), there was no association between alcohol consumption and the outcomes of interest (Table 2). However, the ISS score reached borderline statistical significance between the no alcohol and high-alcohol groups (Table 2), with a trend towards lower ISS in the high-alcohol group when compared with the no alcohol group and the low-alcohol group (*P* = 0.052). Patients in the high-alcohol group were less likely to suffer a severe (ISS > 15) head injury (59.8% versus 68.5%), severe chest injury (48.5% versus 54.8%), and severe extremity injury (1.7% versus 4%), when compared to those in the low-alcohol group (Table 2). Individuals in the high-alcohol group were also less likely to suffer severe head injury (59.8% versus 66.5%), severe chest injury (48.5% versus 56.9%), and severe extremity injury (1.7% versus 5.3%) when compared to those in the no alcohol group (Table 2). 

Table 3 demonstrates adjusted odds ratios (OR) of blood alcohol level for injury severity after controlling for age, gender, MVC characteristic (single vehicle crash versus multiple vehicle crash), location of MVC, injured person type, and seatbelt use. There was no difference in mortality OR 1.09 (0.73–1.62) or severe abdominal injury OR 1.56 (0.98–2.50) between the high-alcohol and low-alcohol groups. Severe head injury OR 0.65 (0.48–0.87), severe chest injury OR 0.58 (0.42–0.80), and severe extremity injury OR 0.10 (0.01–0.76) were less likely to occur in the high-alcohol group compared with the low-alcohol group. When adjusting for coefficients of blood alcohol level for ISS and length of stay, no differences were found among the groups (Table 4).

Experimental studies in animals suggest that alcohol may influence the pathophysiologic response to injury through a variety of proposed mechanisms, such as reduction in cardiac output, alterations in cardiac enzyme metabolism, and disturbances in the response to hemorrhagic shock by altering hemodynamics and the balance of lactic acidosis [33–37]. Unfortunately, clinical and epidemiological studies have provided conflicting results for the relationship between alcohol and severity of injury [26].This may be in part due to confounding variables. For instance, police-reported MVC data in BC suggests that alcohol is significantly associated with an increased risk of fatal injuries, largely due to the fact that alcohol correlates with other risk factors, such as speeding and not wearing seat belts, resulting in more serious crashes and injuries [38]. It has also been suggested that extreme blood alcohol may be associated with increased resource utilization in trauma patients [39]. Swearingen and colleagues demonstrated that positive BAC resulted in increased resource utilization via higher probability of ICU admission and an increased percentage of ICU days highlighting the impact of alcohol consumption in trauma patients on health care systems [39].

Our study failed to demonstrate a correlation between the amount of alcohol consumption, measured via BAC, and mortality or length of hospital stay. In fact, the high-alcohol group demonstrated a 35% reduction in serious head injury, 42% reduction in serious chest injury, and 90% reduction in serious extremity injury when compared with the no alcohol cohort after adjusting for potential confounders (Table 3). This implies that although those who have been under the influence of alcohol may be more likely to be responsible for MVCs [5], they are not more likely to suffer more severe injuries to vital organs such as head and chest injuries when compared to their more sober counterparts. A recent study by Plurad et al. demonstrated that severely injured MVC patients with high BAC have a higher incidence of severe head trauma and septic complications when compared to sober MVC victims, but the high BAC group demonstrated superior adjusted survival rates [32]. Furthermore, Kapur and colleagues found that individuals with a high BAC had a decreased frequency of infections, CNS complications, renal changes, and coagulopathy when compared with those with a negative BAC [40]. Our findings that MVCs are more likely to occur at night, with multiple crash mechanisms and more likely to occur on highways adds to the complexity of factors influencing MVCs, as well as their potential adverse impact on injury severity.

 Strengths of our study include the fact that BC has a centralized Trauma Registry with a comprehensive system for collecting and maintaining data. The BCTR also provides information for individuals who are less likely to have subordinate comorbidities, such as lacerations, animal bites, or chronic subdurals, while including patients with an ISS >12 leading to a reduction of potential complete or partial confounding from these sources. Our analyses also provides data that adjusts for confounders such age which may influence outcomes assessing injury severity and length of hospital stay [41]. Furthermore, Canada has guidelines for accreditation of trauma services throughout the country which decreases the likelihood of treatment factors impacting our assessment on outcomes such as mortality, an outcome that is less prone to bias even in observational studies, although not completely free from possible confounding [42]. Limitations of our study include the fact, as with any observational study, that causation cannot be inferred due to the inherent nature of the study design. When examining the results of serious limb injuries, the event rate was quite low and the confidence interval was wide suggesting that this estimate may lack precision. Alcohol may be the most prevalent single drug detected in drivers admitted as a result of MVC, but there is a possibility of concomitant drug use that may have potentially confounded our results, but we were unable to adjust for this as data was unavailable [43]. We were missing data on seatbelt use in approximately 16% of participants which may have altered some of our estimates. Finally, as with many observational studies, the impact of residual confounding is difficult to ascertain.

## 4. Conclusions

Our study highlights the difficulty to confer a dose-response correlation for alcohol use and injury severity that complements animal experiments. But more importantly, it highlights the need for further research into this area. It is well-established that drinking alcohol before driving is strongly correlated with injury crashes [44]. However, our study and others imply that alcohol consumption does not necessarily predict more serious injuries among those involved in MVCs [11–25]. From a public health perspective, the legal limit of alcohol consumption prior to driving is an important question and the results of our study imply that less is more. For instance, our study implies that those who are less intoxicated are more likely to confer serious injuries from MVCs than those who are more intoxicated. Serious head, chest, and extremity injuries can have debilitating effects on victims of MVCs in terms of their personal and professional lives notwithstanding the impact for increased medical and social supports. Our study also aids clinicians involved in the management of victims of MVCs such that BAC cannot be used as a reliable predictor of injury severity, particularly at the time of triage and preliminary management. In any trauma situation, initial treatment is crucial to management and our study implies that BAC may not be a useful guide to elucidate the most appropriate diagnostic and therapeutic strategies as it is not correlated well with injury severity in victims of MVCs. Having said this, further research is required which may minimize selection, measurement, and confounding biases inherent in health administrative databases. Perhaps a multicentre prospective study that investigates the dose-response relationship of high BAC on injury outcome and extent of resource utilization may be warranted, so that appropriate public health policies and treatment strategies may be developed for frontline clinicians.

## Figures and Tables

**Table 1:** Characteristics of patients and MVC.

		No ETOH Group [%] (*n* = 1,445)	Low ETOH Group [%] (*n* = 273)	High ETOH Group [%] (*n* = 605)	Total [%] (*n* = 2,323)	*P* value*
Age in years	<18	10.1	13.2	7.3	9.7	<0.001
	19–45	47.9	59.3	70.7	55.2	
	46–64	22.9	18.7	19.0	21.4	
	65+	19.1	8.8	3.0	13.7	
Gender	Male	63.3	76.9	82.8	70.0	<0.001
	Female	36.7	23.1	17.2	30.0	
Season	Summer	50.0	53.9	52.2	51.0	NSS
	Winter	50.0	46.2	47.8	49.0	
Time	Night time	41.2	68.3	78.8	53.9	<0.001
	Day time	58.9	31.8	21.2	46.1	
Day	Weekend	30.3	37.7	46.9	35.5	<0.001
	Weekday	69.7	62.3	53.1	64.5	
Location of MVA	Street or highway	95.6	95.7	98.5	96.4	0.007
	Home, place for recreation and sport, public building, or other	4.4	4.3	1.5	3.6	
MVA type	Multiple crash	43.1	31.5	23.2	36.6	<0.001
	Single crash	56.9	68.5	76.8	63.5	
Injured person	Driver	53.1	56.1	53.6	53.6	0.002
	Passenger	23.3	27.9	19.1	22.8	
	Pedestrian	23.6	16.0	27.4	23.7	
Seatbelt	Not used	27.7	44.4	48.3	35.3	<0.001
	Used	58.0	38.5	29.8	47.9	
	Unknown	14.4	17.1	22.0	16.8	

*Comparing the high ETOH group with the low/no ETOH groups.

NSS: not statistically significant.

**Table 2:** Clinical characteristics of patients.

		No ETOH Group (*n* = 1,445)	Low ETOH Group (*n* = 273)	High ETOH Group (*n* = 605)	Total (*n* = 2,323)	*P* value^#^
Severe head injury	%	66.5	68.5	59.8	64.85	0.016
Severe chest injury	%	56.9	54.8	48.5	54.64	0.023
Severe abdomen injury	%	25.3	28.1	28.8	26.44	NSS
Severe extremity injury	%	5.3	4.0	1.7	4.18	0.012
ISS	Mean (SE)	31.3 (0.3)	32.4 (0.7)	30.3 (0.5)	31.2 (0.3)	NSS*
Length of stay (days)	Mean (SE)	22.6 (0.7)	21.3 (1.4)	22.7 (1.4)	22.5 (0.6)	NSS*
Mortality	%	15.5	16.9	13.6	15.15	NSS

^#^Comparing high ETOH group with the low/no ETOH groups.

*ANOVA was performed.

NSS: not statistically significant.

**Table 3:** Adjusted odds ratios of blood alcohol level for injury severity.

	Serious head injury	*P* value	Serious chest injury	*P* value	Serious abdomen injury	*P* value	Serious extremity injury	*P* value	Mortality	*P* value
No	Reference		Reference		Reference		Reference		Reference	
Low	0.82	NSS	0.87	NSS	1.62	NSS	0.69	NSS	1.30	NSS
	(0.55–1.22)		(0.59–1.29)		(0.92–2.87)		(0.20–2.40)		(0.81–2.10)	
High	**0.65 **	<0.05	**0.58 **	<0.05	1.56	NSS	**0.10 **	<0.05	1.09	NSS
	**(0.48**–**0.87)**		**(0.42**–**0.80)**		(0.98–2.50)		**(0.01**–**0.76)**		(0.73–1.62)	

Table 3 compares the low ETOH group with the no ETOH group and high ETOH group with no ETOH group.

*Injured person's age, gender, MVA characteristics (single crash or not), location of MVA, injured person type, and seatbelt use were adjusted.

NSS: not statistically significant.

**Table 4:** Adjusted coefficients of blood alcohol level for ISS and length of stay.

	ISS		Length of stay	
	*β*	*P* value^∧^	*β*	*P* value^∧^
No	reference		reference	
Low	−0.074	NSS	−2.46	NSS
High	−1.387	NSS	−1.58	NSS

*Injured person's age, gender, MVC characteristics (single crash or not), location of MVC, injured person type, and seatbelt use were adjusted.

^∧^Compares low ETOH group with no ETOH and the high ETOH group with no ETOH group.

NSS: not statistically significant.
